# Prenatal and Postnatal Serum PCB Concentrations and Cochlear Function in Children at 45 Months of Age

**DOI:** 10.1289/ehp.1307473

**Published:** 2014-07-22

**Authors:** Todd A. Jusko, Renata Sisto, Ana-Maria Iosif, Arturo Moleti, Sonˇa Wimmerová, Kinga Lancz, Juraj Tihányi, Eva Šovčiková, Beata Drobná, L’ubica Palkovičová, Dana Jurečková, Kelly Thevenet-Morrison, Marc-André Verner, Dean Sonneborn, Irva Hertz-Picciotto, Tomáš Trnovec

**Affiliations:** 1 Division of Epidemiology, Department of Public Health Sciences, University of Rochester School of Medicine and Dentistry, Rochester, New York, USA; 2 Department of Occupational Hygiene, INAIL (Istituto Nazionale per l’Assicurazione contro gli Infortuni sul Lavoro), Monte Porzio Catone, Italy; 3 Division of Biostatistics, Department of Public Health Sciences, School of Medicine, University of California, Davis, Davis, California, USA; 4 Department of Physics, University of Rome Tor Vergata, Rome, Italy; 5 Slovak Medical University, Bratislava, Slovakia; 6 The Štefan Kukura Hospital and Policlinic, Michalovce, Slovakia; 7 Department of Environmental Health, Harvard School of Public Health, Boston, Massachusetts, USA; 8 Institute of Environmental Medicine, Karolinska Institute, Stockholm, Sweden; 9 Division of Environmental and Occupational Health, Department of Public Health Sciences, School of Medicine, University of California, Davis, Davis, California, USA

## Abstract

Background: Some experimental and human data suggest that exposure to polychlorinated biphenyls (PCBs) may induce ototoxicity, though results of previous epidemiologic studies are mixed and generally focus on either prenatal or postnatal PCB concentrations exclusively.

Objectives: Our aim was to evaluate the association between pre- and postnatal PCB concentrations in relation to cochlear status, assessed by distortion product otoacoustic emissions (DPOAEs), and to further clarify the critical periods in development where cochlear status may be most susceptible to PCBs.

Methods: A total of 351 children from a birth cohort in eastern Slovakia underwent otoacoustic testing at 45 months of age. Maternal pregnancy, cord, and child 6-, 16-, and 45-month blood samples were collected and analyzed for PCB concentrations. At 45 months of age, DPOAEs were assessed at 11 frequencies in both ears. Multivariate, generalized linear models were used to estimate the associations between PCB concentrations at different ages and DPOAEs, adjusting for potential confounders.

Results: Maternal and cord PCB-153 concentrations were not associated with DPOAEs at 45 months. Higher postnatal PCB concentrations at 6-, 16-, and 45-months of age were associated with lower (poorer) DPOAE amplitudes. When all postnatal PCB exposures were considered as an area-under-the-curve metric, an increase in PCB-153 concentration from the 25th to the 75th percentile was associated with a 1.6-dB SPL (sound pressure level) decrease in DPOAE amplitude (95% CI: –2.6, –0.5; *p* = 0.003).

Conclusions: In this study, postnatal rather than maternal or cord PCB concentrations were associated with poorer performance on otoacoustic tests at age 45 months.

Citation: Jusko TA, Sisto R, Iosif AM, Moleti A, Wimmerová S, Lancz K, Tihányi J, Šovčíková E, Drobná B, Palkovičová L, Jurečková D, Thevenet-Morrison K, Verner MA, Sonneborn D, Hertz-Picciotto I, Trnovec T. 2014. Prenatal and postnatal serum PCB concentrations and cochlear function in children at 45 months of age. Environ Health Perspect 122:1246–1252; http://dx.doi.org/10.1289/ehp.1307473

## Introduction

Persistent organic pollutants (POPs) are organic compounds of anthropogenic origin that resist degradation and accumulate in the food chain (Agency for Toxic Substances and Disease Registry 2000). Exposure to POPs during critical periods in fetal life may alter the development of the neuroendocrine and other systems (Grandjean and Landrigan 2006). Early exposure to polychlorinated biphenyls (PCBs) in experimental studies produces auditory system impairment (Crofton and Rice 1999; Crofton et al. 2000a, 2000b; Goldey et al. 1995; Herr et al. 1996; Powers et al. 2006). In studies of rats, ototoxicity involves early postnatal exposure to PCBs via lactation, an up-regulation of hepatic uridine diphospho-glucuronosyltransferases, and subsequent hypothyroxinemia during a critical period of cochlear development (Crofton and Zoeller 2005). For instance, distortion product otoacoustic emissions in 18-month-old rodents were reduced in amplitude and thresholds were increased after exposure of the pregnant dams to a PCB mixture (Aroclor 1254) (Lasky et al. 2002).

Previous studies of the association between PCB exposure and measures of hearing in children have produced mixed results. For example, among participants in the Collaborative Perinatal Project, maternal PCB concentration in pregnancy was not associated with sensorineural hearing loss at 8 years of age (Longnecker et al. 2004). In contrast to these null results, higher cord blood PCB concentrations were associated with a higher hearing threshold on audiometry at 7 years of age in a cohort of children in the Faroe Islands (Grandjean et al. 2001). Although both of these studies focused on *in utero* exposure to PCBs, previous studies of children and adolescents living in a highly polluted area of eastern Slovakia showed adverse associations between PCB concentrations measured concurrently with otoacoustic emissions (OAEs) during childhood (Trnovec et al. 2008, 2010). Previously published studies have not examined both *in utero* and postnatal PCB concentrations in relation to measures of child hearing. The repeated measures of PCB concentration in the present study allows examination of multiple exposure time points in relation to hearing outcomes in childhood.

## Materials and Methods

*Study population and follow-up*. This report is based on an ongoing birth cohort study in eastern Slovakia that enrolled 1,134 mother–infant pairs during 2002–2004 (Hertz-Picciotto et al. 2003). Initial participants were recruited from two districts: Michalovce, which has high PCB contamination in the environment from a chemical manufacturing plant (*n* = 812); and Stropkov/Svidnik, located 66 km to the northwest, which has lower environmental levels of PCBs (*n* = 322). Mothers gave informed consent and were enrolled at the time they came to the hospital for delivery. The protocol excluded *a*) mothers with more than four previous births, *b*) mothers < 18 years of age, *c*) mothers who had resided < 5 years in their district, and *d*) mothers with a major illness during pregnancy. Following the births, we also excluded mothers whose infants had severe birth defects. Follow-up occurred at 6 and 16 months of age for the entire cohort, and at 45 months, it was limited to children in the Michalovce district. Thus, no data from the Stropkov/Svidnik district are included in the present analysis. Of the 812 mother–infant pairs initially enrolled from the Michalovce district at birth, 441 (54%) underwent auditory testing at 45 months; the remaining mother–infant pairs declined to participate or were lost to follow-up. The study protocol was approved by institutional review boards at the University of California, Davis, and the Slovak Medical University.

*Exposure assessment*. During the mother’s delivery hospital stay, two 9-mL vacutainer tubes were used to collect maternal blood for PCB and lipid determination, and cord blood was collected just after delivery. The 6-, 16-, and 45-month blood draws took place at the hospital pediatrics department, where up to 9 mL of blood was collected for PCB and lipid analysis. Details on the handling of specimens and the isolation of serum have been presented elsewhere (Jusko et al. 2010; Park et al. 2008). Fifteen PCB congeners [IUPAC (International Union of Pure and Applied Chemistry) numbers 28, 52, 101, 105, 114, 118, 123+149, 138+163, 153, 156+171, 157, 167, 170, 180, and 189] were determined in maternal and infant/child serum samples. The procedure for determination of PCB concentrations involved extraction, cleanup, and quantitation by high-resolution gas chromatography with electron capture detection, as described previously (Conka et al. 2005; Jusko et al. 2010; Kočan et al. 1994). For a portion of 6-month samples (8%), high-resolution mass spectrometry was used to quantitate PCB concentrations (Chovancová et al. 2012). To ensure that analysis type (gas chromatography vs. mass spectrometry) did not bias our 6-month results, we ran an additional 6-month PCB model, which added an indicator variable for method of analysis. The “method-adjusted” PCB hearing estimate was identical to the estimate without adjustment for analysis method, suggesting that the analysis method did not confound the association; thus, this variable was not considered further. Wet-weight concentrations (nanograms per milliliter) were determined at the Department of Toxic Organic Pollutants at the Slovak Medical University in Bratislava. This laboratory serves as the National Reference Laboratory for Dioxins and Related Compounds for the Slovak Republic and has been certified by the Slovak National Accreditation Service (ISO/IEC 17025:2005, certification no. S-111). Further, this laboratory regularly participates in interlaboratory comparison tests, such as the Intercomparison Programme (German External Quality Assessment Scheme) (G-EQUAS 2009) and the Interlaboratory Quality Assessment coordinated by the World Health Organization (2000). Total serum lipids were measured at a commercial laboratory (Alpha Medical, Bratislava, Slovakia) accredited by the Slovak National Accreditation Service (ISO/IEC 15189:2007). Total lipid concentrations were estimated using the enzymatic summation method (Akins et al. 1989).

*Otologic and audiological assessments*. At age 45 months, 441 children initially underwent otological and auditory testing as part of their participation in this study of PCBs, in the Department of Otorhinolaryngology at the Michalovce district hospital. First, an otorhinolaryngologist conducted an otoscopic examination on all children to ensure that the ear was free of infection and obstructions. After examination of the outer and middle ear, tympanometry (GSI 38 Auto Tymp; Grason-Stadler Inc., Milford, NH, USA) was employed as a means of screening children for middle ear function. All tympanometric assessments were conducted in a sound-proof room by the head nurse in the otorhinolaryngology department. Tympanograms were scored based on Jerger’s classification (Jerger 1970), which is a tool for clinical interpretation of middle ear pathology. As a general rule, in children with tympanograms classified as “B,” middle ear pathology may be indicated (e.g., fluid or infection behind the eardrum or a perforation of the eardrum), and proper otoacoustic emissions may not be recorded. For a small proportion of children in our study (7%), both ears were classified as “B.” We nevertheless attempted complete otoacoustic evaluations in all children regardless of Jerger score.

*Distortion product otoacoustic emissions (DPOAEs)*. In the present study we used DPOAEs as an objective measure of hearing function at 45 months of age. OAEs are sounds of cochlear origin, which can be recorded in the ear canal. They are a by-product of a vulnerable active feedback mechanism, located in the cochlear outer hair cells, which contribute greatly to the remarkable sensitivity and frequency discrimination of hearing (Kemp 2002). Because both the OAE response level and the hearing threshold level depend on the effectiveness of the outer hair cell amplification, OAEs can be used as an effective diagnostic tool for detecting hearing loss of cochlear origin. In DPOAE experiments, a stimulus consisting of two nearby frequencies, f_1_ and f_2_, is delivered in the ear canal. The acoustic stimuli are transmitted through the middle ear and reach the cochlea, where they propagate longitudinally as traveling waves along the basilar membrane (BM). Each frequency component f of the stimulus is amplified and absorbed at a characteristic resonant place x(f) on the BM. The nonlinearity of the BM response accounts for the generation of DPOAEs. In the cochlear region, near x(f_2_), that is simultaneously excited by both frequencies, traveling waves are generated at the frequency f_DP_ = 2f_1_ – f_2_ (Shera and Guinan 1999). These waves are transmitted back through the middle ear, and eventually recorded in the ear canal, as DPOAEs. For this reason, the DPOAE signal recorded at the f_DP_ frequency is associated with the cochlear functionality at the characteristic frequency of its generation place (f_2_), both in the data presentation and in the diagnostic interpretation.

*Relationship between DPOAEs and pure-tone audiometry*. When possible, the sensitivity of hearing is directly assessed by pure-tone audiometry, a behavioral technique that measures the hearing threshold, defined as the minimum pure tone level producing perception, at a set of standard frequencies. Unfortunately, this technique requires the active collaboration of the subject (e.g., hand raise, button press, verbal response), and is less reliable when applied to 45-month-old children (Beahan et al. 2012). In adults, DPOAEs are correlated with hearing threshold (as assessed by pure-tone audiometry), as established in several cross-sectional studies (e.g., Bonfils et al. 1988; Gorga et al. 1993; Moulin et al. 1994; Nieschalk et al. 1998; Sisto et al. 2007). As noted by Engdahl et al. (2013), unlike pure-tone audiometry, OAEs are less influenced by inner hair cell function, and they may therefore serve as a more sensitive measure of cochlear function compared with pure-tone audiometry.

*Assessment and analysis of DPOAEs in present study*. Children were examined while sitting in a soundproof room, and the manufacturer’s protocol was followed. For a quiet and cooperative child, the DPOAE measurement took < 10 min. DPOAEs were recorded using the Echoport ILO 292 USB-I (Otodynamics Ltd., Hatfield, Herts., UK), in response to pairs of primary tones of nearby frequencies, f_1_ and f_2_, with f_2_ varied in one-fourth–octave steps between 1,000 and 5,657 Hz, using a constant frequency ratio f_2_/f_1_ = 1.22. Both primary levels were set to 70 dB SPL (decibel sound pressure level). For each frequency step, a signal analyzer picked up the DPOAE response component at the f_DP_ frequency, producing amplitude spectra called DP-grams. The associated noise floor was rather constant across subjects, so the DPOAE signal-to-noise ratio (SNR) was strongly correlated to the response level. Because the detection of hearing impairment is associated with decreased levels of the DPOAE response, rejecting data with low SNR would introduce a serious bias in the data analysis. For this reason, our choice was to include all the data in the analysis, adopting the choice of attributing half the noise amplitude (noise – 6dB) to all signal levels below this threshold. Although the quantitative meaning of DPOAE levels lower than the noise is certainly questionable, our purpose was to avoid the systematic error that is always associated with using an SNR-based data selection criterion when the outcome variable is strongly correlated with SNR itself.

Of the 441 children who initially visited the district hospital for follow-up, DPOAEs were completed on 351 (81%) children. Reasons that the DPOAE could not be conducted or the reading was not valid included the child not staying still or the child having upper respiratory/ear conditions such as acute sinusitis or inflammation of the middle ear/acute tubotympanic catarrh at the time of testing at 45 months. For these 351 children with DPOAE data, 193 had at least one DPOAE frequency measurement in both ears, whereas 93 children had DPOAE measures in the left ear only, and 65 children in the right ear only.

*Measurement of covariates*. After the original enrollment at the time of delivery, trained nursing staff administered a questionnaire during the 5-day hospital stay to obtain information on lifestyle, diet, and living environment, past pregnancies and medical conditions, medication use before and during pregnancy, and sociodemographic data. Romani ethnicity was assigned if the ethnic origin of either of the mother’s parents was Romani, the Romani language was spoken at home, or the mother was planning to raise her child with the Romani language; this definition matched well with additional information, such as the family’s last name. Otherwise, ethnicity was assigned as Slovak/other neighboring European. Other variables, obtained for the 3 months before conception and during pregnancy, included maternal smoking and alcohol use and the mother’s history of illness, including respiratory symptoms, asthma, or allergy. At the 16- and 45-month follow-up visits, mothers again completed questionnaires to update demographic, lifestyle, and dietary information. When the child was 45 months, mothers were also asked questions related to the child’s hearing, whether the child had experienced loud noise, whether any member of the family had experienced permanent hearing loss and, if so, whether the cause was congenital. We also verified that no child was administered potentially ototoxic drugs such as intravenous aminoglycoside antibiotics or vancomycin, antimalarials, or chemotherapeutic agents.

From medical records abstracted at birth, 16, and 45 months, we obtained the child’s birth weight, gestational age (weeks), and weight at well- and sick-child visits with their pediatricians. Of particular concern for this analysis, diagnoses of otitis media between birth and 16 months of age were abstracted.

*Statistical methods*. Selection of PCB congeners. Although we determined the concentrations of 15 PCB congeners in serum specimens, we focused our analyses on PCB-153 for two reasons: *a*) PCB-153 is highly correlated with total PCB concentration in this cohort (Jusko et al. 2010), and *b*) it was detectable in the vast majority of maternal and child specimens, which facilitated comparisons in effect size across time (as opposed to a summed measure with different congeners contributing to the sum at each time point). All maternal, cord, and child PCB-153 concentrations were above the limit of detection (LOD), except for one sample at 6 months and one sample at 16 months. Where PCB-153 concentrations were below the LOD, we used the values as reported by the laboratory. The “postnatal average” PCB-153 concentration was estimated by computing the area under the serum PCB curve (AUC) from 6 through 45 months of age. Dividing the AUC by the 39-month age span yields an average concentration expressed in nanograms per milliliter or nanograms per gram lipid.

Before evaluating the association between PCBs and DPOAE measures, participants in the 45-month follow-up were compared with those in the original Michalovce birth cohort with regard to sociodemographic factors and maternal PCB-153 concentration. Correlations of PCB-153 concentrations across time points were also examined, and results are presented in text. Further, descriptive statistics for the DPOAEs and correlations comparing the right and left ears were computed, along with correlations within each ear across different frequencies, and are also presented in text.

Multivariate model. In most cases, previous research using DPOAEs as measures of ototoxicity have modeled each ear separately as a function of a set of covariates, or simultaneously modeled both ears, ignoring their inherent correlation. In the present analysis, we fit multivariate generalized linear models that accounted for the correlated nature of the data, because of the repeated assessment of the DPOAE for a given child in each ear, and at 11 test frequencies. This hierarchical approach was chosen because it *a*) provides a more powerful analysis by simultaneously evaluating measures of the left and right ears as well as different frequencies measured in each ear, via a single statistical model (e.g., each of our models contained approximately 5–6,000 observations); and *b*) accounts for the complex pattern of within-child correlation (both across frequencies and across ears), to obtain valid estimates of model coefficients and their standard errors. In statistical models, PCB-153 concentrations (nanograms per milliliter) were transformed using the natural log to reduce the influence of extreme values. For each of the six PCB exposure time points (e.g., maternal, cord, 6-month infant, 16-month infant, 45-month child, and postnatal average), we first built a core model, before adding potential confounders, that included the corresponding natural log transformed PCB exposure (nanograms per milliliter), frequency, side, and the interaction between side and frequency. We initially considered interactions between frequency, side, and PCB concentration to allow for possible differences in the PCB–DPOAE associations at specific frequencies for the two ears, but did not observe any meaningful evidence of heterogeneity in the PCB–DPOAE association. Thus, the results presented here include only an interaction between frequency and side in the model. To account for the correlations of the multiple measurements within a child, we used as a covariance structure the direct product of an unstructured matrix (modeling covariance across the two ears) with an autoregressive matrix (modeling covariance across the 11 frequencies). Adjusted models were then constructed by adding confounders to the six core models. All models were implemented using the MIXED procedure in SAS (version 9.3; SAS Institute Inc., Cary, NC, USA).

Selection of potential confounding variables. We initially selected potential confounding variables based on a graphical approach using directed acyclic graphs (DAGs) (Greenland et al. 1999). Separate DAGs were created for each of the six PCB measures. The potential confounding variables chosen for each DAG were dependent on the model, and included ethnicity, lipid concentration, and child’s sex, weight, age, and breastfeeding duration. The constructed DAGs were used to select minimal sufficient adjustment sets, but we found empirically that only adjustment for ethnicity meaningfully changed our estimated associations. In addition to the variables considered in our DAG, we also considered the number of diagnosed episodes of otitis media the child had between birth and 16 months of age, abstracted from medical records, even though this variable may lie on the causal pathway of PCB concentration and DPOAE amplitude, because some literature suggests a positive association between PCB concentrations and otitis media (Chao et al. 1997; Dallaire et al. 2006). We also fit models that excluded children with congenital hearing loss and children whose mothers reported, at 45 months, that the child was exposed to loud noise during childhood. Even with additional adjustment or restriction, we found little difference in the estimated PCB–DPOAE associations; thus, these variables were not considered further, and our adjusted results are presented with adjustment for ethnicity only (Romani vs. Slovak/other European).

## Results

*Descriptive characteristics of the study sample*. Table 1 shows the characteristics of the 351 children who had a DPOAE measured in at least one ear for at least one frequency, and those from Michalovce in the original cohort (*n* = 812). Overall, these two groups were quite similar, except for ethnicity. Romani families were less likely to participate in the hearing study at 45 months compared with Slovak or other European families (comprising 16% of the study sample at 45 months vs. 21% at birth; Table 1) (*p* = 0.02). Maternal PCB-153 concentrations were highly similar among participants in the present study and the entire Michalovce cohort at birth (*p* = 0.99). Gestation length was inversely associated with maternal PCB-153 concentration, whereas maternal age was positively associated with PCB-153 concentration. At 45 months, child PCB-153 concentration was higher among Romani children, and was strongly related to breastfeeding duration. Romani women also reported longer breastfeeding duration compared to Slovak/European women (data not shown).

**Table 1:** Characteristics and PCB-153 concentrations of mothers and children in the Michalovce cohort and those with hearing data at 45 months of age.

Characteristic	Michalovce cohort *n* = 812 [*n* (%)]^*a*^	45-month follow-up *n* = 351 [*n* (%)]^*a*^	*p*-Value^*b*^	Median PCB-153 conc (ng/g lipid)	
				Maternal (*n* = 754)	45-month child (*n* = 351)
Infant sex
Male	423 (52)	175 (50)	0.48	170	135
Female	389 (48)	176 (50)		169	104
Gestation length (weeks)
< 37	18 (2)	3 (1)	0.37	206	89
37–41	761 (94)	333 (95)		171	126
≥ 42	23 (3)	12 (3)		137	58
Missing	10 (1)	3 (1)		92	154
Maternal education (years)
< 12	324 (40)	122 (35)	0.20	167	104
12–16	412 (51)	202 (57)		171	120
> 16	47 (6)	17 (5)		168	213
Missing	29 (3)	10 (3)		171	143
Ethnicity of child
Romani	174 (21)	55 (16)	0.02	158	140
Slovak/eastern European	638 (79)	296 (84)		172	107
Marital status
Married	719 (89)	319 (91)	0.67	169	124
Never married	57 (7)	20 (6)		173	93
Divorced/separated	7 (1)	3 (1)		259	53
Missing	29 (3)	9 (2)		163	160
Maternal age (years)
18 to < 20	68 (8)	27 (8)	0.91	114	126
20–30	614 (76)	266 (76)		160	106
> 30	130 (16)	58 (17)		228	205
Breastfeeding (months)
None	—	12 (3)		175	19
> 0–6	—	190 (54)		170	60
> 6–12	—	52 (15)		156	180
> 12–18	—	56 (16)		167	309
> 18	—	38 (11)		166	365
Missing	—	3 (1)		171	17
Maternal PCB-153 (ng/g lipid)
Mean ± SD (median)	226 ± 225 (170)	226 ± 183 (168)	0.99
conc, concentration. ^***a***^Percentages may not sum to 100 because of rounding. ^***b***^*p*-Value compares the distribution of characteristics in the Michalovce cohort (*n *= 812) with those with hearing data at 45 months (*n *= 351). Chi-square test was used for categorical variables, and *t*-test for quantitative variables (PCB concentrations).					

*PCB concentrations*. Median maternal serum concentrations of PCB-153 among mother–child pairs with complete model data were 169 ng/g lipid [interquartile range (IQR), 117–266 ng/g lipid] (Table 2). Cord blood concentrations tended to be lower than maternal concentrations (median, 130 ng/g lipid). Median child serum concentrations of PCB-153 were 141 ng/g lipid at 6 months of age (IQR, 41–265), 138 ng/g lipid at 16 months of age (IQR, 41–341 ng/g lipid), and 121 ng/g lipid at 45-months of age (IQR, 45–268 ng/g lipid). The median postnatal average PCB-153 concentration, calculated for 291 children with complete PCB concentrations for 6–45 months was 140 ng/g lipid. The Spearman correlations of lipid-adjusted PCB-153 measurements between maternal and cord were strongly correlated [rho (ρ) = 0.91], as were the individual, age-specific postnatal concentrations (0.87 ≤ ρ ≤ 0.93). The postnatal average concentration was strongly correlated with each postnatal measurement (0.93 ≤ ρ ≤ 0.98), but less so with maternal (ρ = 0.34) and cord concentrations (ρ = 0.37) (*p*-values for all correlations were < 0.001).

**Table 2:** Serum PCB-153 concentrations among participants with complete model data for each exposure.

PCB-153 exposure	*n*	Mean	Min	P10	P25	P50	P75	P90	Max
Wet weight (ng/ml)
Maternal	319	2.31	0.43	0.84	1.13	1.68	2.74	4.44	12.87
Cord	334	0.46	0.05	0.14	0.21	0.31	0.52	0.95	4.27
6-month infant	326	1.41	0.01	0.11	0.24	0.84	1.63	3.13	19.55
16-month infant	320	1.56	0.00	0.11	0.26	0.73	1.80	3.44	16.21
45-month child	345	1.21	0.02	0.12	0.23	0.67	1.53	2.71	11.52
Postnatal average	291	1.46	0.05	0.13	0.28	0.81	1.73	3.17	13.63
Per lipid (ng/g)
Maternal	317	226	46	79	117	169	266	445	1,273
Cord	334	184	14	62	84	130	208	367	1,373
6-month infant	323	224	2	16	41	141	265	499	2,641
16-month infant	318	268	1	18	41	138	341	545	3,504
45-month child	344	210	4	21	45	121	268	467	1,919
Postnatal average	291	250	8	22	48	140	304	509	2,435
Abbreviations: Max, maximum; Min, minimum; P, percentile.									

**Figure 1:**
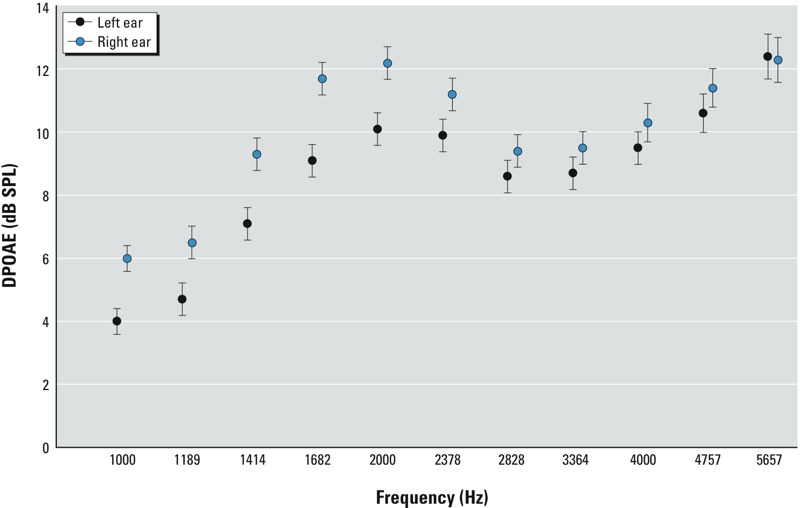
Mean ± SE of distortion product otoacoustic emission (DPOAE) outcomes by frequency, for left (*n* = 286) and right (*n* = 258) ears.

*DPOAE descriptive statistics*. The mean ± SE of DPOAE measures by ear and frequency are shown in Figure 1. Overall, the mean response (decibels SPL) was greater in the right versus the left ear, though this difference tended to decrease as frequencies increased. The variability in DPOAE measures was generally constant across frequencies and ears.

DPOAE measures were positively correlated within an individual. For instance, the Pearson correlation of left with right ear DPOAEs varied from 0.45 to 0.69, depending on the frequency. In all cases, they were statistically significant at the *p* < 0.001 level. Additionally, the Pearson correlation between different frequencies within ears varied from 0.16 to 0.89 (*p* < 0.01 for all frequencies) for the left ear, and from 0.28 to 0.88 (*p* < 0.0001 for all frequencies) for the right ear. Correlations within an ear were higher for DPOAE frequencies that were closer and declined as the frequency differences increased.

*Associations of PCB-153 concentrations with DPOAE measurements*. Overall, Table 3 indicates that postnatal rather than *in utero* (maternal and cord blood) PCB-153 concentrations are associated with adverse DPOAE amplitudes. After adjustment for ethnicity, 6-, 16-, and 45-month PCB-153 concentrations were all inversely associated with DPOAEs assessed at 45 months, with approximately the same magnitude of association (a decrease of about 1 dB SPL as PCB-153 concentration increased from the 25th to 75th percentile). When all postnatal PCB exposures were considered as a cumulative, AUC metric (“postnatal average”), an increase from the 25th to the 75th percentile in postnatal PCB-153 concentration was associated with a 1.57 lower dB SPL (95% CI: –2.61, –0.53; *p* = 0.003). In addition, compared with Slovak/other European children, Romani children had significantly lower DPOAE amplitudes (about 3 dB SPL lower on average) (data not shown).

**Table 3:** Associations between pre- and postnatal PCB-153 concentrations (ng/mL) and 45-month DPOAE amplitudes.

Exposure	*n* (obs)^*a*^	β (95% CI)^*b*^	75th vs. 25th percentile (95% CI)^*c*^	*p*-Value^*d*^
Maternal
Unadjusted	319 (5,504)	0.69 (–0.37, 1.75)	0.61 (–0.33, 1.55)	0.20
Adjusted for ethnicity	319 (5,504)	0.50 (–0.53, 1.53)	0.44 (–0.47, 1.35)	0.34
Cord
Unadjusted	334 (5,713)	0.72 (–0.26, 1.70)	0.66 (–0.24, 1.56)	0.15
Adjusted for ethnicity	334 (5,713)	0.58 (–0.36, 1.53)	0.54 (–0.33, 1.41)	0.22
6-month child PCB-153
Unadjusted	326 (5,559)	–0.52 (–1.04, 0.00)	–0.99 (–1.98, –0.01)	0.05
Adjusted for ethnicity	326 (5,559)	–0.50 (–1.01, 0.02)	–0.94 (–1.93, 0.04)	0.06
16-month child PCB-153
Unadjusted	320 (5,450)	–0.57 (–1.06, –0.08)	–1.11 (–2.05, –0.16)	0.02
Adjusted for ethnicity	320 (5,450)	–0.51 (–0.98, –0.04)	–0.98 (–1.89, –0.08)	0.03
45-month child PCB-153
Unadjusted	345 (5,878)	–0.60 (–1.16, –0.04)	–1.13 (–2.19, –0.07)	0.04
Adjusted for ethnicity	345 (5,878)	–0.56 (–1.12, –0.01)	–1.06 (–2.11, –0.01)	0.05
Postnatal average PCB-153
Unadjusted	291 (4,966)	–0.92 (–1.51, –0.33)	–1.67 (–2.74, –0.60)	0.002
Adjusted for ethnicity	291 (4,966)	–0.86 (–1.44, –0.29)	–1.57 (–2.61, –0.53)	0.003
^***a***^Number of participants in each model, where the reduction from 351 is attributable to missing PCB-153 concentration only; the number of observations (obs) included in each multivariate model is also noted. ^***b***^Estimated change in DPOAE amplitude (dB SPL) for each 1-natural log unit increase in the corresponding PCB-153 exposure (wet weight, ng/mL). ^***c***^Estimated change in DPOAE amplitude (dB SPL) for an increase in the corresponding PCB-153 concentration (wet weight, ng/mL) from the 25th to the 75th percentile. ^***d***^Corresponding *p*-value for each regression model.				

When we adjusted for additional potential confounders (breastfeeding duration, lipid concentration, and child’s age, weight, and sex), the “postnatal average” estimate was not meaningfully different, and was in fact further from the null (results not shown). In addition, we considered the number of episodes of otitis media between birth and 16 months as a potential confounder. History of otitis media was inversely associated with DPOAEs; specifically, as the number of such episodes increased from zero to one, or from one to two or more, DPOAE amplitudes dropped by > 2 dB SPL (data not shown). However, there was no evidence of confounding by otitis media, as we did not observe meaningfully different estimates of the PCB–DPOAE association after adjustment.

## Discussion

In the present study, we observed an inverse association between postnatal serum PCB concentrations and DPOAE amplitudes measured at 45 months. Measures of *in utero* PCB concentration—namely, the maternal and cord blood sample at delivery—were not associated with DPOAE amplitudes at 45 months.

Previous studies from a different group of children from this region have demonstrated cross-sectional associations between child PCB concentrations and hearing impairment at 8–9 years (Trnovec et al. 2008) and at 12 years of age (Trnovec et al. 2010). At 12 years of age, reduced power of transient evoked otoacoustic emissions and DPOAEs were observed at the low frequencies in relation to the child’s current PCB concentrations. These studies examined only cross-sectional associations, and PCB concentrations at 8–9 and 12 years of age are probably less reflective of perinatal exposure; thus, it is unclear whether exposures earlier in life would also be associated with reduced OAE amplitudes. In the Collaborative Perinatal Project conducted in the 1960s in the United States, Longnecker et al. (2004) found no association between prenatal measurements of PCBs measured in maternal serum samples taken during pregnancy and sensorineural hearing loss (based on hearing thresholds) in offspring at 8 years of age. In our analysis, we also did not observe any association with maternal PCB concentration; only the postnatal PCB concentrations were associated with lower DPOAE levels at 45 months of age. On the other hand, higher *in utero* PCB concentrations (as reflected by umbilical cord concentrations) were associated with increased hearing thresholds (Grandjean et al. 2001) in the fish-eating population of the Faroe Islands. However, as noted by Longnecker et al. (2004), PCB concentrations in the Faroese cohort were approximately three times greater than the maternal PCB concentrations in the Collaborative Perinatal Project. A similar magnitude of difference comparing the Faroese cohort with the present study is also apparent (where the Faroese concentrations are approximately three times greater). Thus, it is possible that a threshold for prenatal ototoxicity exists, with maternal/cord concentrations in the Slovak children’s cohort falling below the threshold for an adverse association with cochlear function. In neither the Collaborative Perinatal Project (Longnecker et al. 2004) nor the Faroe Island study (Grandjean et al. 2001) were postnatal PCB concentrations analyzed in relation to hearing thresholds. Because of this, it is unknown whether the Collaborative Perinatal Project would have observed associations similar to those in the present study or not (no association with *in utero* exposure, but with postnatal exposure), or whether postnatal PCB concentrations, in addition to cord blood PCB concentrations, were associated with hearing thresholds in the Faroese cohort.

We also observed that DPOAE measures were generally greater in the right ear compared with the left ear. This finding is consistent with some but not all previous research. For instance, higher DPOAE levels in the right ear have been reported in some studies (Keogh et al. 2001), whereas other studies found no meaningful left–right asymmetry (Pavlovčinová et al. 2010). We did not observe an interaction by ear at 45-months—the relationship between DPOAE measures and PCBs did not differ in the left versus right ear.

The OAE levels are fairly constant through life, at least before the onset of presbyacusia or any other cochlear disturbances (Engdahl et al. 1994; Franklin et al. 1992; Kemp et al. 1986; Prieve et al. 1993). Maturation of the cochlear amplifier in the first months of life is still the object of discussion. At 6 months of age, although anatomical, electrophysiological, and psychoacoustic data suggest full cochlear functional maturity, immaturity of the DPOAE suppression tuning curves has been reported (Abdala et al. 2007), probably also related to developmental changes in the middle ear transmission. Thus, although we observed associations between all the postnatal exposures examined and DPOAE amplitudes, it may be that PCB is ototoxic only in the first few months of life, when exposure to PCBs is greatest as a result of early, exclusive breastfeeding. Specifically, duration of exclusive breastfeeding has been strongly associated with individual and cumulative (AUC) measures of infant serum PCB concentrations in this cohort (Jusko et al. 2012; Trnovec et al. 2011) and others (Ayotte et al. 2003). Moreover, in a study of Inuit infants, Ayotte et al. (2003) modeled 6-month infant plasma concentrations of PCB-153 as a function of maternal PCB-153 concentration and breastfeeding duration. In the model that included only maternal PCB-153 concentration as a predictor, the *R*^2^ value was 0.08; however when breastfeeding duration was added as an additional predictor, the model *R*^2^ was 0.66, strongly suggesting that most of the variance in postnatal PCB concentrations is explained by breastfeeding duration rather than maternal PCB concentrations. Although this study was conducted in a different population, maternal PCB exposures were of similar magnitude, as was the duration of total breastfeeding (Verner et al. 2013). Thus, the adverse associations between 16- and 45-month PCBs and 45-month DPOAE amplitudes may be attributable simply to the strong, positive correlations between postnatal PCB-153 measures over time. On the other hand, there may be continued development of hearing mechanisms beyond 6 months of age, and the results we observed between later PCB measures and 45-month DPOAE amplitudes may be evidence of continued ototoxicity. Interestingly, we observed the strongest adverse associations with DPOAE amplitudes with postnatal average PCB-153 concentration, an exposure metric that takes into account all postnatal PCB measurements using an AUC approach. This may support the hypothesis that duration of PCB exposure, rather than a specific time period, is most critical to hearing development; or it may simply be that the postnatal average metric reduces PCB exposure misclassification and thus effect estimate attenuation toward the null hypothesis. Although a sensitive window for structurally related cochlear impairment would likely originate during prenatal development—in which case the optimal marker would be the PCB concentration in maternal or cord blood—deficits in cochlear function that develop postnatally in infancy or beyond may result from alternative mechanisms, mediated by PCB concentrations measured later. These mechanisms include alteration of intracellular Ca^2+^ signaling via ryanodine receptor–activated Ca^2+^ stores and reactive oxygen species, as discussed by Powers et al. (2006), though these hypotheses may not be extrapolatable to humans.

DPOAE measurements were recorded and analyzed in multivariate models. Notably, the strong correlations of DPOAEs at different frequencies and between the right and left ears dictated that we account for the non-independence of the multiple measurements taken on each child. We accomplished this by fitting generalized multivariate linear models in which the covariances were assumed to be unstructured between ears, and autoregressive according to the frequency tested, based on examination of the pairwise DPOAE correlations across frequencies. This allowed for a single estimate of association across ears and frequency, which used between 5 and 6,000 observations per model to estimate the PCB–DPOAE association, increasing statistical power considerably over analyses that would stratify by frequency and ear.

In the final models for 45-month PCB measurements, we adjusted for ethnicity, and Romani children had significantly lower DPOAE amplitudes (about 3 dB SPL lower on average). In secondary analyses where we adjusted for the number of episodes of otitis media between birth and 16 months, we observed strong inverse associations between a history of these infections and DPOAEs. More specifically, as the number of such episodes increased from zero to one, or from one to two or more, DPOAE amplitudes dropped by > 2 dB SPL. Otitis media has been associated with prenatal PCB exposures (Dallaire et al. 2006), and may therefore function as an intermediate variable. However, inclusion of these infections in the model as a confounder of the PCB–DPOAE relationship did not alter the primary results of interest: The relationship between postnatal PCB level and DPOAE was not altered; nor was it affected by inclusion of breastfeeding duration, congenital hearing loss in another family member, or exposure to loud noise. Overall, the results appear to be quite robust to the choice of control variables, supporting a minimal level of confounding. Putting our results into perspective, although we observed associations between postnatal PCB exposures and DPOAE amplitudes, the adverse PCB association was never greater than the association between ethnicity or ear infections and DPOAE amplitudes.

A major strength of this study is the design, involving multiple PCB measures covering prenatal, infant, and later exposures, which provided the ability to assess potential critical exposure windows. We focused on PCB-153 as the exposure of interest because it is highly correlated with other PCBs in our population and with total PCB concentration, and it is detectable in nearly all samples (Jusko et al. 2010). In fact, PCB-153 comprises approximately 34% (together with di-*ortho*–substituted PCBs 138 and 180, about 80%) of the concentration of the total sum of PCBs in serum in this population. On the other hand, other congeners with different structure–activity relationships (e.g., non-*ortho* substituted dioxin-like PCBs) might show different associations. However, these congeners are not reliably detectable using high-resolution gas chromatography, which is a limitation of our study.

Although we had substantial attrition in our study, participation in the follow-up was not related to PCB exposure. Nonetheless, if loss to follow-up was associated with DPOAEs’ being conditional on postnatal PCB levels, some selection bias could have occurred. Further, we observed greater attrition among Romani families, but inclusion of ethnicity as a covariate should reduce the effects of selection. Although we focused on PCBs, interaction with other ototoxic agents is possible, and is a potential weakness of our study. For instance, in rats, combined exposure to doses of PCBs and polybrominated diphenylethers (PBDEs) that, by themselves, were without effect, did produce significant hearing loss, indicating that PBDEs have the potential to interact with PCBs in producing hearing loss (Poon et al. 2011).

On balance, this study adds further evidence to a growing body of literature in both rodents and humans suggesting cochlear dysfunction with increased exposure to PCBs. The mechanisms for an impact of postnatal PCB exposure versus prenatal PCB exposure remain to be clarified.
